# Amorphous nanosilica induce endocytosis-dependent ROS generation and DNA damage in human keratinocytes

**DOI:** 10.1186/1743-8977-8-1

**Published:** 2011-01-15

**Authors:** Hiromi Nabeshi, Tomoaki Yoshikawa, Keigo Matsuyama, Yasutaro Nakazato, Saeko Tochigi, Sayuri Kondoh, Toshiro Hirai, Takanori Akase, Kazuya Nagano, Yasuhiro Abe, Yasuo Yoshioka, Haruhiko Kamada, Norio Itoh, Shin-ichi Tsunoda, Yasuo Tsutsumi

**Affiliations:** 1 Graduate School of Pharmaceutical Sciences, Osaka University, 1-6 Yamadaoka, Suita, Osaka 565-0871, Japan; 2 Laboratory of Biopharamceutical Research (Pharmaceutical Proteomics), National Institute of Biomedical Innovation, 7-6-8, Saito-Asagi, Ibaraki, Osaka, 567-0085, Japan; 3 The Center for Advanced Medical Engineering and Informatics, Osaka University, 1-6, Yamadaoka, Suita, Osaka, 565-0871, Japan

## Abstract

**Background:**

Clarifying the physicochemical properties of nanomaterials is crucial for hazard assessment and the safe application of these substances. With this in mind, we analyzed the relationship between particle size and the *in vitro *effect of amorphous nanosilica (nSP). Specifically, we evaluated the relationship between particle size of nSP and the *in vitro *biological effects using human keratinocyte cells (HaCaT).

**Results:**

Our results indicate that exposure to nSP of 70 nm diameter (nSP70) induced an elevated level of reactive oxygen species (ROS), leading to DNA damage. A markedly reduced response was observed using submicron-sized silica particles of 300 and 1000 nm diameter. In addition, cytochalasin D-treatment reduced nSP70-mediated ROS generation and DNA damage, suggesting that endocytosis is involved in nSP70-mediated cellular effects.

**Conclusions:**

Thus, particle size affects amorphous silica-induced ROS generation and DNA damage of HaCaT cells. We believe clarification of the endocytosis pathway of nSP will provide useful information for hazard assessment as well as the design of safer forms of nSPs.

## Background

With recent developments in nanotechnology, various kinds of nanomaterials have been designed and produced throughout the world. Nanomaterials have been widely used in consumer and industrial applications, such as medicine, cosmetics and foods, because they exhibit unique physicochemical properties and innovative functions [1]. For example, materials such as amorphous silica nanoparticles (nSPs) and titanium dioxide (TiO_2_) are colorless and reflect ultraviolet light more efficiently than micro-sized particles. Consequently, these substances are already used as functional ingredients in many cosmetics such as foundation creams and sunscreens.

However, concerns over the potentially harmful effects of nanomaterials have been raised precisely because they possess novel properties that are different from those of microsized materials. Increasing numbers of studies show that many types of nanomaterials, such as carbon nanotubes, fullerenes, quantum dots, zinc oxide and TiO_2_, have a harmful effect on cells and rodents [2-14]. For example, previous studies reported that various nanoparticles induced toxicological effects mainly in lung, liver, spleen and kidney tissues [3,10,15-19]. *In vivo *toxicity studies in Sprague Dawley rats showed that inhaled silver nanoparticles elicited chronic inflammation in the lungs [20]. After intravenous injection with silica nanoparticles in BALB/c mice, 70 nm particles induced liver injury at 30 mg/kg, while 300 nm or 1000 nm had no effect [21]. Recent evidence indicates that the small size and high surface area of nanomaterials may cause unpredictable genotoxic properties [22]. For example, induction of DNA damage by gold-, silver-, cobalt-, TiO_2_-nanoparticles has been reported. The results from various studies suggest that these nanomaterials may cause DNA damage by an indirect pathway through promoting oxidative stress and inflammatory responses *via *dysfunction of mitochondria or inflammasomes. Central to the study of nanotoxicology is genotoxicity, the study of genetic aberrations following exposure to nanomaterials, because it is known that an increased genetic instability is associated with the development of cancer.

A sufficient understanding of the relationship between the physicochemical characteristics of nanomaterials governing their cytotoxicity (i.e. genotoxicity) and the identification of factors that influence their associated hazards are essential for the development of safer nanomaterials [22-25]. Since the linkage analysis is the sole methods for developing safe nanomaterials, many researchers have conducted extensive efforts [26-30]. In this context, the aim of our study was to investigate the relationship between particle size and in vitro hazard of amorphous nanosilica (nSP), especially focusing on DNA damage, using human keratinocyte cells.

## Results and Discussion

**Figure 1:**
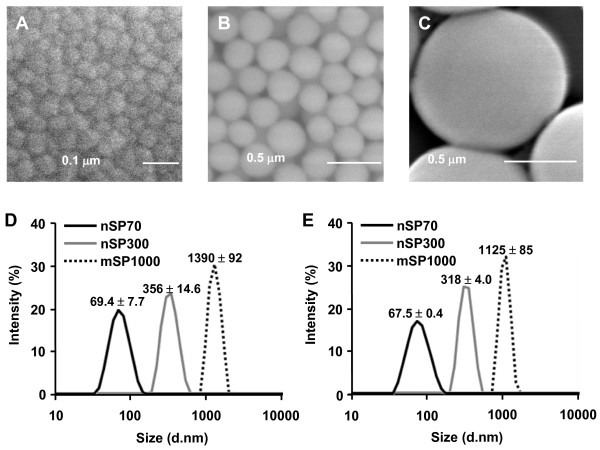
**Scanning electron microscopy (SEM) analysis and spectrum of size distribution of amorphous silica particles**. **(A-C) **SEM photomicrographs of silica particles used in this study: nSP70 (**A**), nSP300 (**B**) and mSP1000 (**C**). Scale bars: 0.1 mm (**A**) and 0.5 mm (**B and C**). **(D and E) **Size distribution of nSP70 (black), nSP300 (gray) and mSP1000 (dashed line) in water (**D**) or PBS (**E**) were measured by a dynamic light scattering method.

We first analyzed the physicochemical properties of the commercially available silica particles of 70, 300 and 1000 nm in diameter (nSP70, nSP300 and mSP1000, respectively). Close examination of the silica particles of different particle sizes (nSP70, nSP300, mSP1000) by scanning electron microscopy (SEM) revealed that all the particles used in this study were spherical and the primary particle sizes were approximately uniform (Figure 1A-C). The size distribution spectrum of each set of silica particles in a neutral solvent showed a single peak. Moreover, the average particle size corresponded almost precisely to the anticipated size for each sample (Figure 1D and 1E). These results suggest that the silica particles used in this study remained as stable well-dispersed particles in solution.

**Figure 2:**
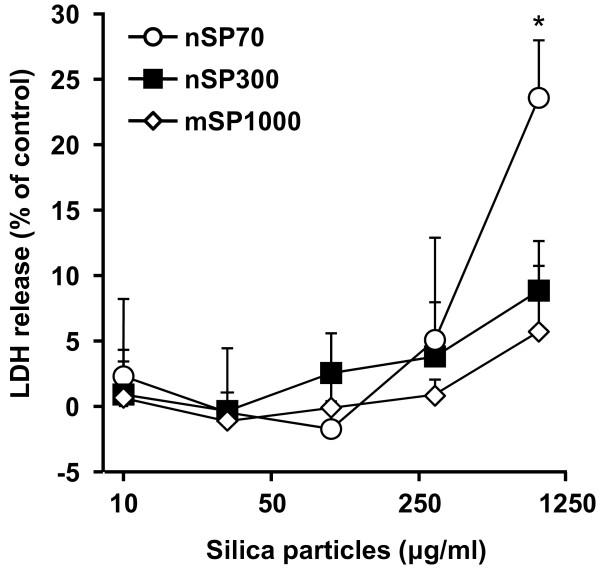
**Effect of silica particles on membrane damage**. Cellular membrane damage in HaCaT cells after incubation with nSP70 (circles), nSP300 (squares) and mSP1000 (diamonds) for 24 h was evaluated by the LDH release assay. The percentage cellular membrane damage was calculated relative to the negative (medium) controls. Data are presented as means ± SD (n = 3).*P < 0.01 vs same dose of nSP300 and mSP1000.

Cosmetic products containing nSP, such as those used in skincare treatments, have been on the market for a considerable period of time. Adult human skin has an average surface area of 1.95 m^2^, weighs 3.18 kg and comprises over 300 million cells. The skin is the largest organ in the human body, which provides protection against heat, cold, electromagnetic radiation and chemical damage. Indeed, skin cells are likely to have the highest frequency of exposure to nSPs. Hence, a safety evaluation of nSPs using dermal cells is essential. Based on this consideration, using the HaCaT human keratinocyte cell line as a model system, we studied the effects of various sized silica particles on cell function. Specifically, we used HaCaT cells to perform the LDH release assay to assess membrane damage induced by silica particles. We found that membrane damage was not observed in nSP300- and mSP1000-treated HaCaT cells. By contrast, LDH release increased after exposure of the cells to nSP70 in a dose-dependent manner (Figure 2). This observation suggested that membrane damage in keratinocytes increased significantly when the particle size was less than 100 nm. The decrease of particle size changes the physicochemical properties of the silica particles, such as surface area and the number of functional groups per particle weight, which are both increased [31-34]. In addition, subsequent experiments were performed at a non-toxic dose (less than 300 μg/ml) in order to exclude the toxic effects of nSP70.

**Figure 3:**
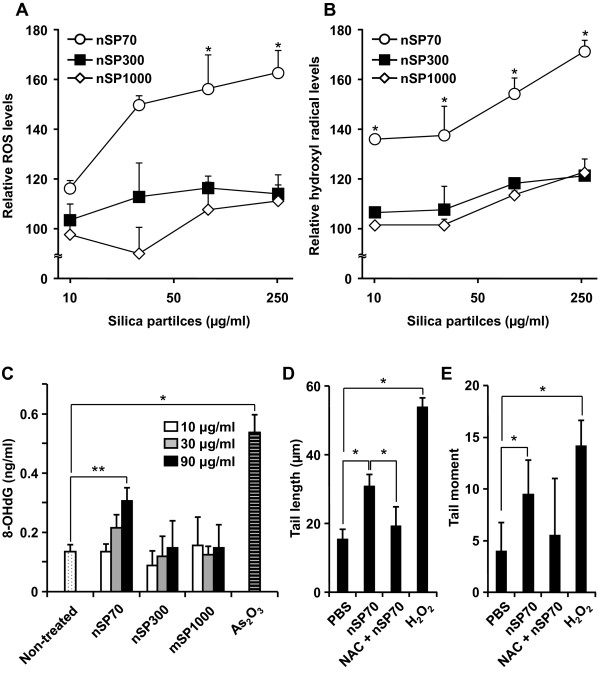
**Detection of oxidative stress induced by silica particle treatment in HaCaT cells**. Detection of total ROS and hydroxyl radical induced by silica particle treatment in HaCaT cells. HaCaT cells were incubated with various concentrations of nSP70 (circles), nSP300 (squares), and mSP1000 (diamonds) for 3 h. (**A**) Total ROS induced by treatment with silica particles were expressed as relative fluorescence units in the DCFH assay.*P < 0.01 vs same dose of nSP300 and mSP1000. (**B**) Hydroxyl radical was measured by hydroxyphenyl fluorescein (HPF) assay. Data shown are means ± SD (n = 3).*P < 0.01 vs same dose of nSP300 and mSP1000. (**C**) Detection of 8-OH-dG induced by silica particle treatment in HaCaT cells. HaCaT cells were incubated with 10, 30 or 90 mg/ml nSP70, nSP300, or mSP1000, and As_2_O_3 _(positive control) for 3 h. Data shown are means ± SD (n = 3). *P < 0.01, **P < 0.05. (**D and E**) Effects of ROS inhibitor on DNA strand breaks induced by silica particle treatment in HaCaT cells. HaCaT cells were pretreated with 2 mM N-acetylcystein (NAC) for 30 min (NAC + nSP70) or nSP70 alone, prior to incubation with 90 mg/ml nSP70 for 3 h. As a positive control, HaCaT cells were treated with 0.2 mM H_2_O_2 _for 3 h. (**D**) Column height shows the tail length. (**E**) Column height shows the tail moment. Data shown are means ± SD of at least 16 cells per sample. Results shown are representative of more than three independent experiments. *P < 0.01.

Some reports have indicated that intracellular generation of reactive oxygen species (ROS) is induced by nSP [35-37]. Furthermore, it has recently been reported that crystalline silica induces intracellular ROS generation *via *NADPH oxidase activation following uptake by endocytosis [38,39]. Based on these reports, ROS generation and DNA damage are an obvious means of assessing the hazard posed by nSP. Firstly, total intracellular ROS generation was measured in silica particle-treated HaCaT cells using 2'7'-dichlorodihydorofluorescein diacetate (DCFH-DA). Silica particles of all sizes were found to induce intracellular ROS generation in a dose-dependent fashion (Figure 3A). However, ROS generation by nSP70 treatment was significantly greater compared with nSP300 and mSP1000 treatment at the same particle concentration. Additionally, we confirmed that hydroxyl radicals, one of the most highly reactive ROS, were generated in HaCaT cells treated with silica particles, in particular with nSP70 (Figure 3B). Even in the 10 μg/ml-treated group, hydroxyl radical-generation effects of nSP70-treatment were 1.4 times higher than that of nSP300 and mSP1000-treated groups. These results suggested that silica particle-induced intracellular ROS generation was significantly increased by decreasing the particle size to less than 100 nm. ROS are defined as either "primary" or "secondary". Primary ROS (e.g. superoxide, O_2_^-^) can be generated through metabolic processes or through the activation of oxygen, which results in the formation of a reactive nucleophilic molecule of oxygen i.e., superoxide anion. These reactive species may interact with other molecules, such as redox active transition metals (e.g. iron) or enzymes, resulting in the production of "secondary" ROS (e.g. ^•^OH), which are primary mediators of DNA damage. Consequently, we analyzed the formation of 7'8'-dihydro-8-oxodeoxyguanosine (8-OH-dG) as an indicator of ROS-induced DNA damage. When HaCaT cells were treated with various concentrations of silica particles for 3 h, 8-OH-dG levels in nSP300- and mSP1000-treated cells remained constant regardless of silica particle dose and were equal to the levels found in untreated cells (Figure 3C). By contrast, 8-OH-dG levels increased upon exposure of the cells to nSP70 in a dose-dependent manner. After treatment with nSP70 at 90 μg/ml the level of 8-OH-dG increased significantly compared with non-treated cells.

8-OH-dG is known as a major index of oxidative DNA damage related to mutagenesis, carcinogenesis and the aging process [40,41]. These reports, together with our results, suggest the possibility that nSP70 may be carcinogenic. Moreover, nSP-induced ROS may induce genotoxicity *via *DNA strand breaks, oxidative DNA damage and mutation. Indeed, DNA damage was detected in nSP70-treated HaCaT cells. In addition, nSP70-mediated DNA damage was inhibited by pre-treatment with the ROS scavenger, N-acetylcystein (NAC) (Figure 3D and 3E). From the results of the present study, we suggest that ROS play an important role in cellular responses such as nSP-induced DNA damage. However, the reason why ROS generation varies with particle size has not yet been clarified.

**Figure 4:**
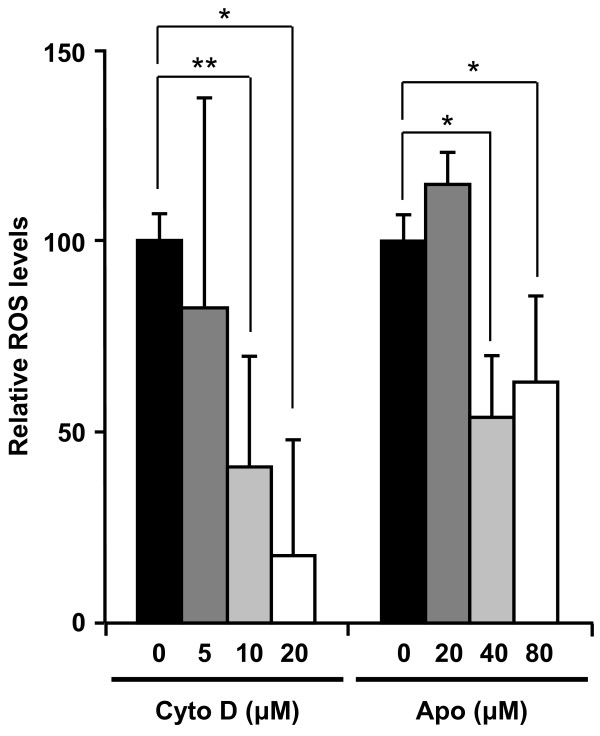
**Effects of endocytosis and NADPH oxidase inhibitor on generation of ROS induced by silica particle treatment**. HaCaT cells were pretreated with cytochalasin D or apocynin for 30 min prior to incubation with 270 mg/ml nSP70 for 3 h. ROS induced by silica particle treatment were expressed as relative fluorescence units, which means that ROS intensity of each silica particle alone and non-treatment is 100 and 0 respectively, in the DCFH assay. Data shown are means ± SD (n = 3). *P < 0.01, **P < 0.05.

**Figure 5:**
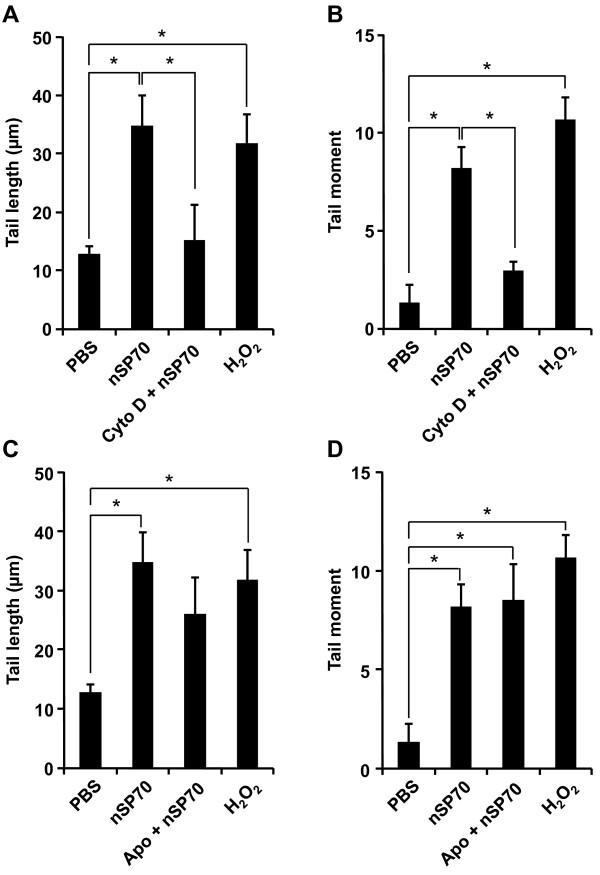
**Effects of endocytosis and NADPH oxidase inhibitor on DNA damage by silica particle treatment**. Effects of endocytosis inhibitor (**A and B**) or NADPH oxidase inhibitor (**C and D**) on DNA strand breaks induced by silica particle treatment in HaCaT cells. (**A and B**) HaCaT cells were pretreated with 10 mM cytochalasin D (Cyto D) for 30 min (Cyto D + nSP70) or nSP70 alone, prior to incubation with 90 mg/ml nSP70 for 3 h. (**C and D**) HaCaT cells were pretreated with 40 mM apocynin (Apo) for 30 min (Apo + nSP70) or nSP70 alone, prior to incubation with 90 mg/ml nSP70 for 3 h. As a positive control, HaCaT cells were treated with 0.2 mM H_2_O_2 _for 3 h. (**A and C**) Column height shows the tail length. (**B and D**) Column height shows the tail moment. Data shown are means ± SD of at least 16 cells per sample. Results shown are representative of more than three independent experiments. *P < 0.01.

Fine or ultrafine particulate matter (PM), such as diesel exhaust particles or crystalline silica, often induces ROS generation that contributes to the induction of DNA damage or apoptosis. Although the mechanisms underlying the PM-induced oxidative stress response remains unclear, strong evidence supports PM phagocytosis as a stimulus for increased oxidative stress *via *NADPH oxidase activation [38,42,43]. In addition, Walee Chamulitrat *et al*. reported that HaCaT cells constitutively express Nox components Rac1, p40phox, and p67phox proteins [44]. In HaCaT skin keratinocyte cells, stimuli such as epidermal growth factor, Ca^2+^-ionophore A23187, lysophosphatidic acid are capable of producing ROS [45-47]. Thus, one potential candidate for the nSP70-mediated DNA damage is ROS, which is produced by NADPH oxidase upon nSP70 phagocytosis. In order to assess the relationship between the uptake pathway and ROS generation, we measured the production of ROS induced by nSP70 in the presence or absence of a specific inhibitor of endocytosis. After treatment with cytochalasin D, an inhibitor of actin polymerization [48], ROS generation induced by nSP70 was measured by DCFH-DA assay. Results indicated that ROS generation induced by nSP70 was inhibited by pretreatment with cytochalasin D in a dose-dependent manner (Figure 4). Furthermore, nSP70-induced DNA damage was also significantly reduced by pretreatment with cytochalasin D (Figure 5A and 5B). These findings suggest that the silica particles entered the cells mainly through actin-mediated endocytosis, such as the macropinocytosis pathway, thereby inducing ROS generation and DNA damage. It is well-known that NADPH oxidase, which exists in the cytosol, cellular membrane and subcellular compartment membranes, becomes activated and generates ROS after ingestion of microorganisms into the phagosome and/or endosome [49-51]. Moreover, it is reported that TiO_2 _particles induce IL-1ß production by NADPH oxidase-mediated ROS generation in the human macrophage cell line [52]. Likewise, NADPH oxidase exists in the cytosol and membranes of non-phagocyte cells, including HaCaT cells [44]. Additionally, it had been reported that inflammasomes are activated by actin-mediated endocytosis of crystalline silica, which lead to NADPH oxidase activation and ROS generation [38,39,53]. Consequently, in order to determine the role of NADPH oxidase in silica particle-induced ROS generation, the effects of pretreatment with the NADPH oxidase inhibitor, apocynin, a well-known NOX inhibitor [49,54], were investigated. As expected, nSP70-induced ROS generation was inhibited in the presence of apocynin (Figure 4). In contrast, DNA damage induced by nSP70 was not inhibited by pretreatment with apocynin (Figure 5C and 5D). Taken together, these results suggest that nSP70-mediated DNA damage was induced by ROS generated by an unknown mechanism, and not *via *NADPH oxidase. Nox1 activation may initiate large bursts of ROS that can mediate the killing of pathogens, such as *H. pylori *[55]. Thus, NOX1 activation has been implicated in the cutaneous innate immunity to bacterial infections of the skin. A more detailed evaluation of the mechanism that underlies nSP70-mediated NOX activation is essential. Nonetheless, based on our results and the work of others, we speculate that nSP70s are treated almost like pathogens by HaCaT cells.

A number of mechanisms underlie the ability of nanoparticles to cause DNA damage. As mentioned above, a key mechanism that is often described is the ability of particles to cause the production of ROS [32,56]. One possible mechanism of particle-mediated DNA damage is the ability of particles to stimulate target cells to produce oxidants/genotoxic compounds e.g., by affecting mitochondrial electron transport, activation of NADPH oxidase, or inducing cytochrome P450 enzymes. Our results show that nSP70-mediated DNA damage of HaCaT cells occurred *via *a mechanism that did not involve NADPH oxidase. Alternatively, transition metal ions (such as cadmium, chromium, cobalt, copper, iron, nickel, titanium and zinc) released from certain nanoparticles have the potential to cause the conversion of cellular oxygen metabolic products such as H_2_O_2 _and superoxide anions to hydroxyl radicals, which is one of the primary DNA damaging species. Well-known examples of the consequences of metal ion-contamination in relation to nanotoxicity have been described for carbon nanotubes. Indeed, iron contaminants in CNT have been shown to result in a substantial loss of glutathione and increased lipid peroxidation in alveolar macrophages, indicators of oxidative stress [57]. However, our data suggests that the nSPs used in this study, nSP70, nSP300 and mSP1000, were not contaminated with metal ions (data not shown). Thus, it is highly unlikely that metal ion contamination is involved in nSP70-induced DNA damage. Another hypothesis is that the size of nSPs is related to its oxidative stress. As particle size decreases, the particle unit of mass and overall surface area increases. This larger surface area enhances catalytic activity. Indeed, it has been widely reported that increased surface area of these particles increases reactivity because surface atoms have a tendency to possess high energy bonds. In order to gain stabilization, these surface bonds will readily react with other molecules [58]. The specific surface area was calculated by means of the following equation; s = 6/dρ (where s, specific surface area (m^2^/g); ρ, density (g/cc); d, diameter (μm)). The specific surface area of nSP70, nSP300 and mSP1000 calculated using this equation was 43, 10 and 3 m^2^/g, respectively. When specific area is considered, rather than particle concentration, the membrane damage activity of nSP70 and nSP300-treated cells shows almost the same level of LDH release per unit surface area (data not shown). In terms of ROS generation and DNA oxidation, nSP70 is more potent than nSP300. These results suggest that nSP70, which possesses a larger specific surface area compared to the counterpart micron-sized silica particles, has a much greater chance of interaction with biomolecules. Consequently, nSP70 causes direct cellular damage and promotion of oxidative stress. In addition to these hypotheses, nanoparticles may gain direct access to DNA *via *nuclear transport. However, this mechanism seems very unlikely given that the nuclear pore complex is known to be 8-30 nm in diameter, depending on cell type [59]. Nonetheless, some studies have reported that nanoparticles can penetrate the nuclear membrane, such as silica nanoparticles (40-70 nm) [60]. Detailed analysis of the mechanism of DNA damage induced by nanoparticles is currently underway. This information will be a critical determinant in the design of safer nSPs and will provide valuable information for hazard assessment of nSPs.

Here, we report the effects induced by well-dispersed amorphous silica particles (nSPs) on human keratinocyte (HaCaT) cells. In addition to our own work, other studies have shown that well-dispersed nSPs induce cytotoxicity, including LDH release, in a dose-dependent and size-dependent manner using a macrophage cell line [61,62]. On the other hands, Lin et al. reported that nSPs mediated cytotoxicity/DNA damage against A549 cells were not correlated with particle size [36]. Further, Barnes et al. reported that nSP induce no genotoxicity in fibroblast 3T3-L1 cells[63]. From the viewpoint of nSP-mediated toxicity, there is no consistency in these four reports including our findings. As mentioned above, there are a number of examples in the literature of conflicting results regarding nSPs. It has becoming increasingly evident that the physicochemical properties of nanomaterials, such as the size, shape, surface charge, fabricating method, etc, play a central role in governing their cellular uptake and subsequent physiologic consequences. Furthermore, experimental conditions, such as cell type and incubation time, are critical for the nanotoxicologic studies. Hence, given the inconsistencies it is difficult to draw the same conclusions. However, our results using well-dispersed nSPs indicated that nSPs were more cytotoxic and genotoxic against the human keratinocyte cell line HaCaT.

## Conclusions

In this study, we show that nSP induce certain cellular responses, such as ROS generation and DNA damage. By contrast, their bulk-sized counterparts display a much reduced response. These different responses might be partly due to different mechanisms, such as intracellular uptake and ROS generation. We speculated that receptor-mediated uptake was involved in these phenomena and set out to identify the physicochemical properties that affect receptor endocytosis. We believe a detailed analysis of nSP-internalization will be invaluable for both hazard assessment and the design of safe nSPs.

## Materials and methods

### Silica particles

Suspensions of fluorescent (red-F)-labeled amorphous silica particles (Micromod Partikeltechnologie GmbH) (25 mg/ml and 50 mg/ml) were used in this study; particle size diameters were 70, 300 and 1000 nm (designated as nSP70, nSP300 and mSP1000, respectively). Silica particle suspensions were stored in the dark at room temperature. The suspensions were sonicated for 5 min and then vortexed for 1 min immediately prior to use.

### Cell Culture

The HaCaT human keratinocyte cell line was kindly provided by Dr. Inui [64], Osaka University. HaCaT cells were cultured in Dulbecco's modified Eagle's medium (D-MEM) supplemented with 10% heat-inactivated fetal bovine serum and 0.2 mM L-glutamine. The cells were grown in a humidified incubator at 37°C (95% room air, 5% CO_2_).

### Physicochemical examinations of silica particles

Silica particle suspensions were diluted to 0.25 mg/ml (nSP70), 0.5 mg/ml (nSP300 and mSP1000) with water or PBS, respectively and the average particle sizes were then measured using the Zetasizer Nano-ZS (Malvern Instruments Ltd). The mean size and the size distribution of silica particles were measured by a dynamic light scattering method. The size and shape of silica particles were determined using scanning electron microscopy (SEM). Each silica particle suspension was dropped on the sample stage and dried. The dried silica particles were then observed by SEM.

### LDH release assay

Lactate dehydrogenase (LDH) is released from HaCaT cells exposed to nSP70, nSP300 or mSP1000. The LDH activity of the supernatant of the culture medium was determined using a commercial LDH cytotoxicity test (WAKO, Japan) according to the manufacturer's instructions. In brief, 5 × 10^3 ^cells were seeded into each well of a 96-well plate. After 24 h incubation, cells were treated with nSP70, nSP300, mSP1000 or 0.2% Tween 20 (positive control). After a further 24 h incubation period, 50 μl of medium overlying cells was used for LDH analysis. Absorption of light at 560 nm was measured using a spectrophotometer.

### Detection of Reactive Oxygen Species (ROS)

The generation of total intracellular ROS was measured by monitoring the increasing fluorescence of 2'7'-dichlorofluorescein (DCF). The cell-permeant 2'7'-dichlorodihydorofluorescein diacetate (DCFH-DA; Sigma, St. Louis, MO) enters the cell where intracellular esterases cleave off the diacetate group. The resulting DCFH is retained in the cytoplasm and oxidized to DCF by ROS. Hydroxyl radical was measured by monitoring the increasing fluorescence of hydroxyphenyl fluorescein (HPF; SEKISUI MEDICAL Co., Ltd., Japan). 3 × 10^4 ^HaCaT cells were seeded into each well of a 96-well plate. After 24 h incubation, cells were treated with nSP70, nSP100, nSP300, mSP1000 or 2 mM H_2_O_2 _(positive control). Cells were then washed once with phenol red-free medium, and incubated in 100 μl working solution of DCFH-DA or HPF (10 μM) at 37°C for 30 min. Using the fluorescence reader (ARVO MX; Perkin Elmer, Waltham, MA), the fluorescence of DCF or HPF was monitored at the excitation and emission wavelengths of 485 nm and 530 nm or 490 nm and 515 nm, respectively.

### 8-Hydroxy-2-deoxyguanosine (8-OH-dG) measurement

HaCaT cells were seeded on a 100 mm dish. After 24 h, cells were treated with various concentrations of nSP70, nSP300, mSP1000, 0.2 mM H_2_O_2 _(positive control) or PBS (negative control). After 3 h, cellular DNA was isolated using DNeasy tissue kit (QIAGEN, Germany). Ten μg of DNA was converted to single stranded DNA by incubation with 180 U Exonuclease III (Takara Biotech., Japan) at 37°C for 1 h. The DNA was heated at 95°C for 5 min, rapidly chilled on ice, and digested to nucleosides by incubation with 0.6 U nuclease P1 (Takara) at 37°C for 1 h followed by treatment with 0.6 U *E. coli *alkaline phosphatase (Takara) for a further 1 h. The reaction mixture was centrifuged (6000 × g for 1 min) and the supernatant used for the 8-OHdG assay. The amount of 8-OHdG was measured according to the protocol of the competitive ELISA kit (8-OHdG check; Japan Institute for the Control of Aging, Japan).

### Effects of inhibitor of ROS, endocytosis or NADPH oxidase on DNA strand breaks induced by silica particles

3 × 10^4 ^HaCaT cells were pretreated with 2 mM N-acetylcystein (NAC, ROS scavenger), 10 mM cytochalasin D (endocytosis inhibitor) or 40 mM apocynin (NADPH oxidase inhibitor) for 30 min prior to incubation with 90 mg/ml of nSP70 for 3 h. As a positive control, HaCaT cells were treated with 0.2 mM H_2_O_2 _for 3 h. DNA strand breaks were detected by alkaline comet assay according to the Comet Assay Kit (Trevigen, Gaithersburg, MD). The samples were processed according to the protocol provided in the kit. Twenty-five cells on each slide, randomly selected by fluorescence microscopy, were then analyzed using the Comet Analyzer (Youworks Corporation, Japan).

### Effects of inhibitor of endocytosis, NADPH oxidase or endosomal acidification on generation of ROS induced by silica particles

HaCaT cells were pretreated with various concentration of cytochalasin D (Merck Ltd., Germany) for 30 min prior to incubation with 270 mg/ml nSP70 for 3 h. ROS induced by treatment with silica particles were expressed as relative fluorescence units in the DCFH-DA assay as described above.

### Statistical analysis

Statistical comparisons between groups were performed by one-way ANOVA and a Bonferroni *post hoc *test. The level of significance was set at *P *< 0.05.

## Competing interests

The authors declare that they have no competing interests.

## Authors' contributions

HN and TY designed the study. HN, KM, YN, STo, SK, TH and TA performed experiments. HN and TY collected and analysed data. HN and TY wrote the manuscript. KN, YA, YY, HK, NI and STs gave technical support and conceptual advice. YT supervised the all of projects. All authors discussed the results and commented on the manuscript.

## References

[B1] Salata O Applications of nanoparticles in biology and medicine J Nanobiotechnology 2004 2 3 10.1186/1477-3155-2-3 15119954 PMC419715

[B2] Aiso S Yamazaki K Umeda Y Asakura M Takaya M Toya T Koda S Nagano K Arito H Fukushima S Pulmonary Toxicity of Intratracheally Instilled Multiwall Carbon Nanotubes in Male Fischer 344 Rats Ind Health 2010 in press 10.2486/indhealth.ms1129 20616469

[B3] Chen J Dong X Zhao J Tang G In vivo acute toxicity of titanium dioxide nanoparticles to mice after intraperitioneal injection J Appl Toxicol 2009 29 330 337 10.1002/jat.1414 19156710

[B4] Geys J Nemmar A Verbeken E Smolders E Ratoi M Hoylaerts MF Nemery B Hoet PH Acute toxicity and prothrombotic effects of quantum dots: impact of surface charge Environ Health Perspect 2008 116 1607 1613 10.1289/ehp.11566 19079709 PMC2599752

[B5] Heng BC Zhao X Xiong S Ng KW Boey FY Loo JS Toxicity of zinc oxide (ZnO) nanoparticles on human bronchial epithelial cells (BEAS-2B) is accentuated by oxidative stress Food Chem Toxicol 2010 48 1762 1766 10.1016/j.fct.2010.04.023 20412830

[B6] Kocbek P Teskac K Kreft ME Kristl J Toxicological Aspects of Long-Term Treatment of Keratinocytes with ZnO and TiO(2) Nanoparticles Small 2010 6 1908 1917 10.1002/smll.201000032 20677183

[B7] Liu S Xu L Zhang T Ren G Yang Z Oxidative stress and apoptosis induced by nanosized titanium dioxide in PC12 cells Toxicology 2010 267 172 177 10.1016/j.tox.2009.11.012 19922763

[B8] Moos PJ Chung K Woessner D Honeggar M Cutler NS Veranth JM ZnO particulate matter requires cell contact for toxicity in human colon cancer cells Chem Res Toxicol 2010 23 733 739 10.1021/tx900203v 20155942

[B9] Murray AR Kisin E Leonard SS Young SH Kommineni C Kagan VE Castranova V Shvedova AA Oxidative stress and inflammatory response in dermal toxicity of single-walled carbon nanotubes Toxicology 2009 257 161 171 10.1016/j.tox.2008.12.023 19150385

[B10] Park EJ Kim H Kim Y Yi J Choi K Park K Carbon fullerenes (C60s) can induce inflammatory responses in the lung of mice Toxicol Appl Pharmacol 2010 244 226 233 10.1016/j.taap.2009.12.036 20064541

[B11] Poland CA Duffin R Kinloch I Maynard A Wallace WA Seaton A Stone V Brown S Macnee W Donaldson K Carbon nanotubes introduced into the abdominal cavity of mice show asbestos-like pathogenicity in a pilot study Nat Nanotechnol 2008 3 423 428 10.1038/nnano.2008.111 18654567

[B12] Shin JA Lee EJ Seo SM Kim HS Kang JL Park EM Nanosized titanium dioxide enhanced inflammatory responses in the septic brain of mouse Neuroscience 2010 165 445 454 10.1016/j.neuroscience.2009.10.057 19892005

[B13] Takagi A Hirose A Nishimura T Fukumori N Ogata A Ohashi N Kitajima S Kanno J Induction of mesothelioma in p53+/- mouse by intraperitoneal application of multi-wall carbon nanotube J Toxicol Sci 2008 33 105 116 10.2131/jts.33.105 18303189

[B14] Yamashita K Yoshioka Y Higashisaka K Morishita Y Yoshida T Fujimura M Kayamuro H Nabeshi H Yamashita T Nagano K Carbon nanotubes elicit DNA damage and inflammatory response relative to their size and shape Inflammation 2010 33 276 280 10.1007/s10753-010-9182-7 20174859

[B15] Chen Z Meng H Xing G Chen C Zhao Y Jia G Wang T Yuan H Ye C Zhao F Acute toxicological effects of copper nanoparticles in vivo Toxicol Lett 2006 163 109 120 10.1016/j.toxlet.2005.10.003 16289865

[B16] Duan Y Liu J Ma L Li N Liu H Wang J Zheng L Liu C Wang X Zhao X Toxicological characteristics of nanoparticulate anatase titanium dioxide in mice Biomaterials 2010 31 894 899 10.1016/j.biomaterials.2009.10.003 19857890

[B17] Li JJ Muralikrishnan S Ng CT Yung LY Bay BH Nanoparticle-induced pulmonary toxicity Exp Biol Med (Maywood) 2010 235 1025 1033 20719818 10.1258/ebm.2010.010021

[B18] Li N Duan Y Hong M Zheng L Fei M Zhao X Wang J Cui Y Liu H Cai J Spleen injury and apoptotic pathway in mice caused by titanium dioxide nanoparticules Toxicol Lett 2010 195 161 168 10.1016/j.toxlet.2010.03.1116 20381595

[B19] Liang G Yin L Zhang J Liu R Zhang T Ye B Pu Y Effects of subchronic exposure to multi-walled carbon nanotubes on mice J Toxicol Environ Health A 2010 73 463 470 10.1080/15287390903523378 20391125

[B20] Sung JH Ji JH Yoon JU Kim DS Song MY Jeong J Han BS Han JH Chung YH Kim J Lung function changes in Sprague-Dawley rats after prolonged inhalation exposure to silver nanoparticles Inhal Toxicol 2008 20 567 574 10.1080/08958370701874671 18444009

[B21] Nishimori H Kondoh M Isoda K Tsunoda S Tsutsumi Y Yagi K Silica nanoparticles as hepatotoxicants Eur J Pharm Biopharm 2009 72 496 501 10.1016/j.ejpb.2009.02.005 19232391

[B22] Aillon KL Xie Y El-Gendy N Berkland CJ Forrest ML Effects of nanomaterial physicochemical properties on in vivo toxicity Adv Drug Deliv Rev 2009 61 457 466 10.1016/j.addr.2009.03.010 19386275 PMC2743376

[B23] Hoshino A Fujioka K Oku T Suga M Sasaki FY Ohta T Yasuhara M Suzuki K Yamamoto K Physicochemical Properties and Cellular Toxicity of Nanocrystal Quantum Dots Depend on Their Surface Modification Nano Letters 2004 4 2163 2169 10.1021/nl048715d

[B24] Morishige T Yoshioka Y Inakura H Tanabe A Yao X Narimatsu S Monobe Y Imazawa T Tsunoda S Tsutsumi Y The effect of surface modification of amorphous silica particles on NLRP3 inflammasome mediated IL-1beta production, ROS production and endosomal rupture Biomaterials 2010 31 6833 6842 10.1016/j.biomaterials.2010.05.036 20561679

[B25] Sohaebuddin SK Thevenot PT Baker D Eaton JW Tang L Nanomaterial cytotoxicity is composition, size, and cell type dependent Part Fibre Toxicol 2010 7 22 10.1186/1743-8977-7-22 20727197 PMC2936333

[B26] Fourches D Pu D Tassa C Weissleder R Shaw SY Mumper RJ Tropsha A Quantitative nanostructure-activity relationship modeling ACS Nano 2010 4 5703 5712 10.1021/nn1013484 20857979 PMC2997621

[B27] Puzyn T Leszczynska D Leszczynski J Toward the development of "nano-QSARs": advances and challenges Small 2009 5 2494 2509 10.1002/smll.200900179 19787675

[B28] Shaw SY Westly EC Pittet MJ Subramanian A Schreiber SL Weissleder R Perturbational profiling of nanomaterial biologic activity Proc Natl Acad Sci USA 2008 105 7387 7392 10.1073/pnas.0802878105 18492802 PMC2396702

[B29] Tropsha A Golbraikh A Predictive QSAR modeling workflow, model applicability domains, and virtual screening Curr Pharm Des 2007 13 3494 3504 10.2174/138161207782794257 18220786

[B30] Weissleder R Kelly K Sun EY Shtatland T Josephson L Cell-specific targeting of nanoparticles by multivalent attachment of small molecules Nat Biotechnol 2005 23 1418 1423 10.1038/nbt1159 16244656

[B31] Borm P Klaessig FC Landry TD Moudgil B Pauluhn J Thomas K Trottier R Wood S Research strategies for safety evaluation of nanomaterials, part V: role of dissolution in biological fate and effects of nanoscale particles Toxicol Sci 2006 90 23 32 10.1093/toxsci/kfj084 16396841

[B32] Nel A Xia T Madler L Li N Toxic potential of materials at the nanolevel Science 2006 311 622 627 10.1126/science.1114397 16456071

[B33] Rahman IA Vejayakumaran P Sipaut SC Ismail J Chee KC Size-dependent physicochemical and optical properties of silica nanoparticles Materials Chemistry and Physics 2009 114 328 332 10.1016/j.matchemphys.2008.09.068

[B34] Xia T Kovochich M Brant J Hotze M Sempf J Oberley T Sioutas C Yeh JI Wiesner MR Nel AE Comparison of the abilities of ambient and manufactured nanoparticles to induce cellular toxicity according to an oxidative stress paradigm Nano Lett 2006 6 1794 1807 10.1021/nl061025k 16895376

[B35] Eom HJ Choi J Oxidative stress of silica nanoparticles in human bronchial epithelial cell, Beas-2B Toxicol In Vitro 2009 23 1326 1332 10.1016/j.tiv.2009.07.010 19602432

[B36] Lin W Huang YW Zhou XD Ma Y In vitro toxicity of silica nanoparticles in human lung cancer cells Toxicol Appl Pharmacol 2006 217 252 259 10.1016/j.taap.2006.10.004 17112558

[B37] Wang F Gao F Lan M Yuan H Huang Y Liu J Oxidative stress contributes to silica nanoparticle-induced cytotoxicity in human embryonic kidney cells Toxicol In Vitro 2009 23 808 815 10.1016/j.tiv.2009.04.009 19401228

[B38] Dostert C Petrilli V Van Bruggen R Steele C Mossman BT Tschopp J Innate immune activation through Nalp3 inflammasome sensing of asbestos and silica Science 2008 320 674 677 10.1126/science.1156995 18403674 PMC2396588

[B39] Hornung V Bauernfeind F Halle A Samstad EO Kono H Rock KL Fitzgerald KA Latz E Silica crystals and aluminum salts activate the NALP3 inflammasome through phagosomal destabilization Nat Immunol 2008 9 847 856 10.1038/ni.1631 18604214 PMC2834784

[B40] Ames BN Dietary carcinogens and anticarcinogens. Oxygen radicals and degenerative diseases Science 1983 221 1256 1264 10.1126/science.6351251 6351251

[B41] Harman D The aging process Proc Natl Acad Sci USA 1981 78 7124 7128 10.1073/pnas.78.11.7124 6947277 PMC349208

[B42] Li Z Hyseni X Carter JD Soukup JM Dailey LA Huang YC Pollutant particles enhanced H2O2 production from NAD(P)H oxidase and mitochondria in human pulmonary artery endothelial cells Am J Physiol Cell Physiol 2006 291 C357 365 10.1152/ajpcell.00365.2005 16571865

[B43] Wang T Chiang ET Moreno-Vinasco L Lang GD Pendyala S Samet JM Geyh AS Breysse PN Chillrud SN Natarajan V Garcia JG Particulate matter disrupts human lung endothelial barrier integrity via ROS- and p38 MAPK-dependent pathways Am J Respir Cell Mol Biol 2010 42 442 449 10.1165/rcmb.2008-0402OC 19520919 PMC2848737

[B44] Chamulitrat W Stremmel W Kawahara T Rokutan K Fujii H Wingler K Schmidt HH Schmidt R A constitutive NADPH oxidase-like system containing gp91phox homologs in human keratinocytes J Invest Dermatol 2004 122 1000 1009 10.1111/j.0022-202X.2004.22410.x 15102091

[B45] Goldman R Moshonov S Zor U Generation of reactive oxygen species in a human keratinocyte cell line: role of calcium Arch Biochem Biophys 1998 350 10 18 10.1006/abbi.1997.0478 9466814

[B46] Goldman R Moshonov S Zor U Calcium-dependent PAF-stimulated generation of reactive oxygen species in a human keratinocyte cell line Biochim Biophys Acta 1999 1438 349 358 10366777 10.1016/s1388-1981(99)00066-9

[B47] Sekharam M Cunnick JM Wu J Involvement of lipoxygenase in lysophosphatidic acid-stimulated hydrogen peroxide release in human HaCaT keratinocytes Biochem J 2000 346 Pt 3 751 758 10.1042/0264-6021:3460751 10698703 PMC1220909

[B48] Sampath P Pollard TD Effects of cytochalasin, phalloidin, and pH on the elongation of actin filaments Biochemistry 1991 30 1973 1980 10.1021/bi00221a034 1899622

[B49] Lambeth JD NOX enzymes and the biology of reactive oxygen Nat Rev Immunol 2004 4 181 189 10.1038/nri1312 15039755

[B50] Li Q Zhang Y Marden JJ Banfi B Engelhardt JF Endosomal NADPH oxidase regulates c-Src activation following hypoxia/reoxygenation injury Biochem J 2008 411 531 541 10.1042/BJ20071534 18397177 PMC3597079

[B51] Ushio-Fukai M Localizing NADPH oxidase-derived ROS Sci STKE 2006 2006 re8 10.1126/stke.3492006re8 16926363

[B52] Morishige T Yoshioka Y Tanabe A Yao X Tsunoda S Tsutsumi Y Mukai Y Okada N Nakagawa S Titanium dioxide induces different levels of IL-1beta production dependent on its particle characteristics through caspase-1 activation mediated by reactive oxygen species and cathepsin B Biochem Biophys Res Commun 2010 392 160 165 10.1016/j.bbrc.2009.12.178 20059972

[B53] Fubini B Hubbard A Reactive oxygen species (ROS) and reactive nitrogen species (RNS) generation by silica in inflammation and fibrosis Free Radic Biol Med 2003 34 1507 1516 10.1016/S0891-5849(03)00149-7 12788471

[B54] Meyer JW Schmitt ME A central role for the endothelial NADPH oxidase in atherosclerosis FEBS Lett 2000 472 1 4 10.1016/S0014-5793(00)01397-1 10781793

[B55] Kawahara T Kuwano Y Teshima-Kondo S Kawai T Nikawa T Kishi K Rokutan K Toll-like receptor 4 regulates gastric pit cell responses to Helicobacter pylori infection J Med Invest 2001 48 190 197 11694959

[B56] Schins RP Mechanisms of genotoxicity of particles and fibers Inhal Toxicol 2002 14 57 78 10.1080/089583701753338631 12122560

[B57] Kagan VE Tyurina YY Tyurin VA Konduru NV Potapovich AI Osipov AN Kisin ER Schwegler-Berry D Mercer R Castranova V Shvedova AA Direct and indirect effects of single walled carbon nanotubes on RAW 264.7 macrophages: role of iron Toxicol Lett 2006 165 88 100 10.1016/j.toxlet.2006.02.001 16527436

[B58] Oberdorster G Geleinr R Johnston C Mercer P Corson N Finkelstein J Ambient ultra fine particles: Inducers of acute Lung injury? Relationships between respiratory disease and exposure to air pollution 1998 ILSI Press, Washington, DC 216 229

[B59] Terry LJ Shows EB Wente SR Crossing the nuclear envelope: hierarchical regulation of nucleocytoplasmic transport Science 2007 318 1412 1416 10.1126/science.1142204 18048681

[B60] Chen M von Mikecz A Formation of nucleoplasmic protein aggregates impairs nuclear function in response to SiO2 nanoparticles Exp Cell Res 2005 305 51 62 10.1016/j.yexcr.2004.12.021 15777787

[B61] Lison D Thomassen LC Rabolli V Gonzalez L Napierska D Seo JW Kirsch-Volders M Hoet P Kirschhock CE Martens JA Nominal and effective dosimetry of silica nanoparticles in cytotoxicity assays Toxicol Sci 2008 104 155 162 10.1093/toxsci/kfn072 18400775

[B62] Waters KM Masiello LM Zangar RC Tarasevich BJ Karin NJ Quesenberry RD Bandyopadhyay S Teeguarden JG Pounds JG Thrall BD Macrophage responses to silica nanoparticles are highly conserved across particle sizes Toxicol Sci 2009 107 553 569 10.1093/toxsci/kfn250 19073995 PMC2639757

[B63] Barnes CA Elsaesser A Arkusz J Smok A Palus J Lesniak A Salvati A Hanrahan JP Jong WH Dziubaltowska E Reproducible comet assay of amorphous silica nanoparticles detects no genotoxicity Nano Lett 2008 8 3069 3074 10.1021/nl801661w 18698730

[B64] Inui S Lee YF Haake AR Goldsmith LA Chang C Induction of TR4 orphan receptor by retinoic acid in human HaCaT keratinocytes J Invest Dermatol 1999 112 426 431 10.1046/j.1523-1747.1999.00548.x 10201524

